# Outcomes of Metabolic and Bariatric Surgery in Populations with Obesity and Their Risk of Developing Colorectal Cancer: Where Do We Stand? An Umbrella Review on Behalf of TROGSS—The Robotic Global Surgical Society

**DOI:** 10.3390/cancers17040670

**Published:** 2025-02-17

**Authors:** Aman Goyal, Christian Adrian Macias, Maria Paula Corzo, Daniel Tomey, Sachin Shetty, Victor Peña, Halil Bulut, Adel Abou-Mrad, Luigi Marano, Rodolfo J. Oviedo

**Affiliations:** 1 Department of General Surgery, Mahatma Gandhi Medical College and Research Institute, Pondicherry-Cuddalore Rd., ECR, Pillayarkuppam 607402, Puducherry, India; doc.aman.goyal@gmail.com; 2 Adesh Institute of Medical Sciences and Research, Bathinda 151109, Punjab, India; 3 School of Medicine, Universidad Catolica de Santiago de Guayaquil, Guayaquil 090615, Ecuador; 4 Department of Health and Science, Hillsborough Community College, Tampa, FL 33614, USA; 5 Center for Space Emerging Technologies (C-SET), Lima 15046, Peru; 6 Department of Surgery, Universidad de Los Andes, Bogota 111711, Colombia; mp.corzo10@uniandes.edu.co; 7 Department of Surgery, Houston Methodist Hospital, Houston, TX 77030, USA; datomey@houstonmethodist.org; 8 University of Texas Health Science Center at San Antonio, San Antonio, TX 78229, USA; shettys@uthscsa.edu; 9 Department of Surgery, HCA Florida Kendall Hospital, Miami, FL 33175, USA; vicopg95@gmail.com; 10 Cerrahpasa School of Medicine, Istanbul University Cerrahpasa, 34098 Istanbul, Turkey; halilibrahim.bulut@ogr.iuc.edu.tr; 11 Department of Surgery, Centre Hospitalier Universitaire d’Orléans, 45100 Orléans, France; adel.abou-mrad@orange.fr; 12 Department of Medicine, Academy of Applied Medical and Social Sciences-AMiSNS: Akademia Medycznych I Spolecznych Nauk Stosowanych, 82-300 Elbląg, Poland; 13 Department of General Surgery and Surgical Oncology, “Saint Wojciech” Hospital, “Nicolaus Copernicus” Health Center, 80-462 Gdańsk, Poland; 14 Department of Surgery, Nacogdoches Medical Center, Nacogdoches, TX 75965, USA; 15 Tilman J. Fertitta Family College of Medicine, University of Houston, Houston, TX 77021, USA; 16 Department of Surgery, Sam Houston State University College of Osteopathic Medicine, Conroe, TX 77304, USA

**Keywords:** metabolic bariatric surgery, colorectal cancer, obesity, cancer prevention, systematic review

## Abstract

**Introduction**: Obesity is a chronic disease associated with increased risk for several cancers, including colorectal cancer (CRC), a leading cause of cancer-related mortality. The majority of CRC cases are associated with modifiable risk factors. Metabolic and bariatric surgery (MBS) is a proven, durable, and successful intervention for obesity. This study aimed to evaluate the impact of MBS on CRC risk through measures of association, such as relative risk (RR) and odds ratio (OR). **Methods**: A systematic search of PubMed, Scopus, Web of Science, ScienceDirect, and Embase was conducted to identify systematic reviews (SR) and meta-analyses examining the relationship between obesity treated with MBS and CRC incidence. The PICO framework guided inclusion criteria, and three independent reviewers screened articles using Rayyan software. Quality assessment was performed using AMSTAR2. **Results**: Of 1336 screened articles, 10 SR met inclusion criteria, encompassing 53,452,658 patients. Meta-analyses consistently showed a significant reduction in CRC risk following MBS in patients with severe obesity. Risk reductions were reported by Liu et al. (RR: 0.46, 95% CI: 0.32–0.67, *p* < 0.01), Chierici et al. (RR: 0.46, 95% CI: 0.28–0.75, *p* = 0.018), Wilson et al. (RR: 0.69, 95% CI: 0.53–0.88, *p* = 0.003), and Pararas et al. (RR: 0.56, 95% CI: 0.40–0.80, *p* < 0.001). Sensitivity analyses supported these findings. For colon cancer, Liu and Chierici both reported an RR of 0.75 (95% CI: 0.46–1.21, *p* = 0.2444) with significant heterogeneity (I^2^ = 89%). A trend towards reduced rectal cancer risk (RR: 0.74, 95% CI: 0.40–1.39, *p* = 0.3523) was noted but limited by fewer studies. Sex-specific analyses revealed protective effects in both sexes, with a more pronounced impact in females (RR: 0.54, 95% CI: 0.37–0.79, *p* = 0.0014). **Conclusions**: This umbrella review synthesizes current evidence on the impact of MBS on CRC risk, highlighting a consistent protective association. The findings also indicate a potential risk reduction for both colon and rectal cancer, with a more pronounced effect observed among females compared to males. Given the profound implications of MBS on cancer incidence, morbidity, and mortality, further high-quality, long-term studies are essential to deepen our understanding and optimize its role in cancer prevention and patient care.

## Simple Summary

Obesity is a major public health concern and is linked to an increased risk of colorectal cancer (CRC), one of the leading causes of cancer-related deaths. Many cases of CRC are preventable through lifestyle changes and medical interventions. Metabolic bariatric surgery (MBS) is a well-established treatment for severe obesity and may also help lower the risk of developing CRC. This study reviewed existing research to assess whether MBS reduces CRC risk. The findings indicate that patients undergoing MBS experience a significant reduction in CRC risk, with particularly strong benefits observed in women. There is also evidence suggesting a potential reduction in risks for both colon and rectal cancers. These results highlight the importance of MBS not only for weight management but also as a potential strategy for cancer prevention. More long-term research is needed to fully understand its impact on cancer prevention and patient outcomes.

## 1. Introduction

Obesity remains a significant global health concern. The World Health Organization (WHO) reported that, as of 2022, roughly one in eight people globally were affected by obesity [1]. Notably, obesity rates among adults have doubled since 1990, while those among adolescents have increased fourfold [2]. The escalating prevalence of obesity across diverse populations and age groups has heightened concern about its short- and long-term implications, prompting extensive medical research [3].

Obesity is now recognized as a complex, multifactorial chronic disease with far-reaching consequences beyond its physical manifestations. It contributes to a wide array of comorbidities, including diabetes mellitus, dyslipidemia, metabolic dysfunction associated fatty liver disease, and obstructive sleep apnea [4]. Increasing evidence underscores the link between obesity and the development, progression, and mortality associated with various cancers, such as colorectal, postmenopausal breast, endometrial, kidney, and esophageal cancers [5].

The association between obesity and cancer risk is well-documented. The International Agency for Research on Cancer (IARC) working group has established sufficient evidence linking higher body fat levels to increased risks of several cancers, including colorectal cancer (RR: 1.3, 95% CI: 1.3–1.4), pancreatic cancer (RR: 1.5, 95% CI: 1.2–1.8), and renal cell carcinoma (RR: 1.8, 95% CI: 1.7–1.9) [6]. Additionally, epidemiological data suggest that a 30% to 70% increased risk of CRC can be attributed to obesity [7]. Obesity is not only associated with an increased risk of cancer but may also increase the risk of cancer recurrence and mortality in cancer survivors [8].

Glover et al. (2019) conducted a retrospective cross-sectional study analyzing a total of 34 million individuals, of whom 8.8 million were aged 20–39 years. The analysis, limited to adults aged 20–39 years, identified 1700 cases of early-onset colorectal cancer. Comparing early-onset colorectal cancer with non-early-onset colorectal cancer individuals, obesity was associated with a significantly increased risk of early-onset colorectal cancer, with an OR of 1.819 (95% CI: 1.618–2.044; *p* < 0.001) [9].

Colorectal cancer (CRC) is particularly concerning, as it is the third most diagnosed cancer and the second leading cause of cancer-related deaths worldwide [10]. Even more troubling is the rise in early-onset CRC, particularly in high-income countries such as the United States, Canada, and Australia. However, the lack of global data limits the ability to evaluate trends comprehensively [11].

Given the alarming global obesity rates, medical interventions for obesity management have gained prominence. Among these, metabolic and bariatric surgery (MBS) has emerged as a highly effective and durable treatment option for severe obesity and its associated comorbidities. Long-term studies have demonstrated the efficacy of MBS in inducing substantial and sustained weight loss, improving metabolic parameters and achieving remission of chronic conditions such as type 2 diabetes mellitus and hypertension [12,13,14]. Furthermore, MBS has been shown to reduce the incidence of several cancers and serve as a bridge to other interventions, such as arthroplasty and organ transplantation [12].

The objective of this umbrella review is to synthesize the current evidence on the relationship between obesity treated with MBS and its potential impact on CRC. Although the protective effect of MBS on CRC has been evaluated in previous studies, this umbrella review provides a comprehensive aggregation of data, consolidating findings across diverse populations. This study not only bridges gaps in the existing literature but also offers novel insights into the nuanced impact of individual MBS procedures, thereby contributing to the optimization of cancer prevention strategies.

## 2. Materials and Methods

### 2.1. Search Strategy

We systematically searched five databases: PubMed, Web of Science, Scopus, ScienceDirect, and Embase to identify systematic reviews and meta-analyses that evaluated the association between obesity treated with MBS and CRC. We registered the protocol of this systematic review in the International Prospective Register of Systematic Reviews (PROSPERO) under the number CRD42024619870. It was conducted according to the Preferred Reporting Items for Systematic Review and Meta-Analysis Protocols (PRISMA-P 2020) guidelines [15]. The search strategy included terms related to obesity, MBS, and CRC. The complete search terms and strategy are provided in the Supplementary Materials (Table S1). Searches were conducted from database inception to 23rd October 2024, with no restrictions on geographic location. Duplicate records were identified and removed using Rayyan software (https://www.rayyan.ai/) [16]. In addition to database searches, gray literature, including dissertations and reports, was excluded, and this decision was based on the scope of our study focusing on peer-reviewed evidence.

### 2.2. Eligibility Criteria

We applied the PICO framework to define the inclusion criteria:

1. Population: adult patients (>18 years); animal studies were excluded.
2. Intervention: MBS (e.g., gastric banding, sleeve gastrectomy, Roux-en-Y gastric bypass).
3. Comparison: CRC.
4. Outcome: association with colorectal cancer, with effect sizes reported as odds ratio (OR), relative risk (RR), or hazard ratio (HR).

Additional inclusion criteria for this study were systematic reviews or meta-analyses evaluating the association between MBS and CRC, as well as articles published in English. Studies were excluded if they were abstracts, editorials, narrative reviews, conference proceedings, or preprints.

### 2.3. Data Extraction

Three reviewers independently screened titles and abstracts using Rayyan software [16]. Full texts of eligible studies were reviewed, and disagreements were resolved by consensus. Data were extracted independently by the same reviewers and cross-checked for accuracy. The following data points were collected:

1. Study identifiers: author names, title, and publication year.
2. Population characteristics: total number of participants and demographic details (if available).
3. Outcomes: effect sizes (OR, RR, HR) and corresponding 95% confidence intervals (CI) for the following:
  - CRC (overall).
  - Colon and rectal cancer (separately).
  - CRC stratified by sex (male and female).
  - CRC associated with specific bariatric surgery types (gastric band, sleeve gastrectomy, Roux-en-Y gastric bypass).

### 2.4. Assessment of Methodological Quality

The quality of systematic reviews included in this umbrella review was rigorously assessed using the A Measurement Tool to Assess Systematic Reviews, Version 2 (AMSTAR 2) [17]. AMSTAR 2 is a reliable and validated tool developed to critically assess the methodological quality of systematic reviews focused on healthcare interventions. It incorporates 16 items, including seven critical domains that significantly impact the reliability and validity of systematic reviews. These critical domains include protocol registration, adequacy of the literature search, justification for excluded studies, risk of bias from individual studies, and appropriate statistical methods for synthesis.

Each systematic review was assigned a quality rating based on AMSTAR 2, ranging from “High Quality” to “Critically Low Quality”. Reviews were classified as High Quality if they met all critical criteria without any non-critical weaknesses. Moderate Quality was assigned to reviews with no critical flaws but some non-critical weaknesses. Low Quality and Critically Low Quality ratings were given to reviews with at least one critical flaw, with the latter reserved for reviews exhibiting multiple significant methodological issues. The evaluation also involved detailed scrutiny of each review’s adherence to AMSTAR 2 standards and documentation of specific flaws or strengths. Discrepancies in AMSTAR 2 ratings were resolved through consensus.

### 2.5. Statistical Analysis

For data synthesis, we employed a qualitative approach followed by quantitative analysis where appropriate. The results of the included studies were pooled using random-effects models to account for variability in study design, sample characteristics, and outcome measures. Overall effect sizes were expressed as RR with 95% confidence intervals (CIs). Heterogeneity between studies was assessed using the I^2^ statistic, with values greater than 50% indicating substantial heterogeneity.

Forest plots were generated using R software (version 4.4.2), employing the meta and metafor packages to visualize pooled estimates and heterogeneity. These plots provided a graphical representation of effect sizes for each study and the overall pooled effect size with its confidence intervals. Statistical significance was determined at a *p*-value of <0.05. All analyses adhered to PRISMA guidelines to ensure methodological rigor and transparency.

A publication bias assessment was conducted using funnel plots and Egger’s test to evaluate small-study effects. The funnel plot was constructed by plotting the standard error against the log risk ratio for each study included in the meta-analysis. The symmetry of the plot was visually inspected to assess potential bias. Additionally, Egger’s regression test was applied to statistically evaluate asymmetry, with a *p*-value <0.05 considered indicative of significant publication bias. All statistical analyses were performed using R (R Foundation for Statistical Computing, Vienna, Austria).

## 3. Results

### 3.1. Study Selection

**Figure 1:**
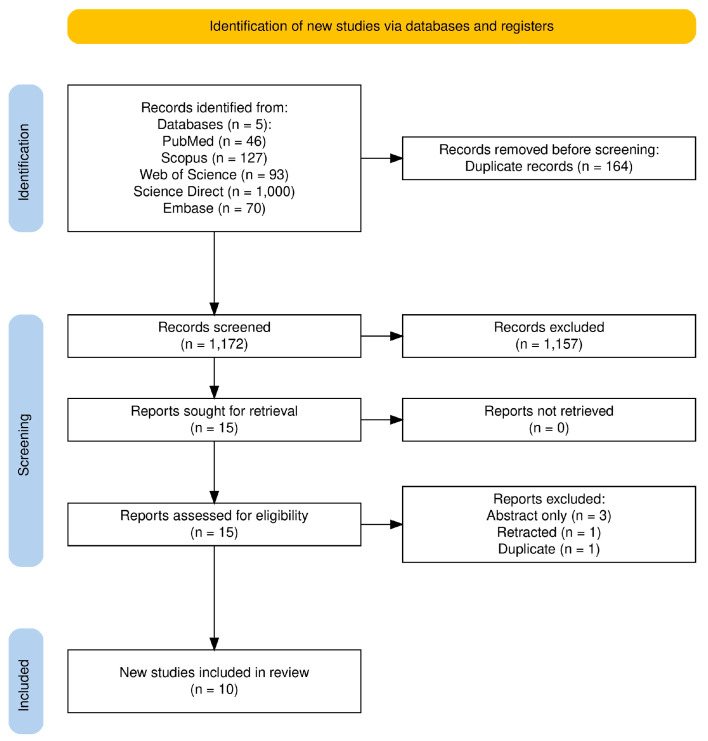
PRISMA flow diagram outlining the screening, inclusion, and exclusion process for studies included in this umbrella review [26].

From the systematic search across five databases, a total of 1336 potentially eligible articles were identified: PubMed (46), Scopus (127), Web of Science (93), ScienceDirect (1000), and Embase (70). After removing 164 duplicate records, 1172 unique articles were screened based on their abstracts using the Rayyan platform. Of these, 1157 articles were excluded after title and abstract screening due to irrelevance or non-compliance with inclusion criteria. A full-text review was conducted for the remaining 15 articles. Following this step, five articles were excluded for the following reasons: one was a duplicate, one had been retracted, and three were available only as abstracts (Table S2). In total, 10 articles fulfilled the inclusion criteria and were included in the umbrella review. Nine of the ten articles were published from 2020 to 2023 [7,18,19,20,21,22,23,24,25], with a median year of 2023. The study selection process is visually represented in Figure 1, generated using the web application developed by Haddaway et al. [26].

### 3.2. Study Characteristics

Two articles originated from the USA (20%), while the remaining correspond to China, Australia, Ireland, Greece, France, Poland, Kuwait, and the UK, each with one single study (10%). The total number of studies included per review ranged from 4 to 32. Chierici A et al. [23] had the highest number of patients across their studies (12,517,893), followed by Liu YN et al. [18] (12,497,322) and Clapp B et al. [7] (6,279,722) (Table 1). All studies included were meta-analyses. The overlapping articles in the included studies is shown in Table S3.

### 3.3. CRC Risk Reduction Following MBS

The studies consistently demonstrated a significant reduction in CRC risk following MBS in populations with severe obesity. RR was the most frequently reported effect size, with eight studies [18,19,21,22,23,24,25,27], while OR was used in two studies [7,20].

Seven out of eight studies reported statistically significant RR. For instance, Liu et al. (2023) [18] reported the most pronounced reduction in CRC risk (RR = 0.46, 95% CI: 0.32–0.67, *p* < 0.01) in a cohort exceeding 12 million patients from China. Similarly, Chierici et al. (2023) [23] from France observed a significant risk reduction (RR = 0.46, 95% CI: 0.28–0.75, *p* = 0.018). Other notable findings included Wilson et al. (2023) [19] from Australia (RR = 0.69, 95% CI: 0.53–0.88, *p* = 0.003) and Pararas et al. (2023) [22] from Greece (RR = 0.56, 95% CI: 0.40–0.80, *p* < 0.001).

Two studies using OR also supported the protective association. Davey et al. (2023) [20] reported an OR of 0.53 (95% CI: 0.36–0.77, *p* < 0.001) in over six million patients in Ireland, while Clapp et al. (2022) [7] from the USA observed a reduced CRC risk (OR = 0.64, 95% CI: 0.49–0.84).

**Figure 2:**
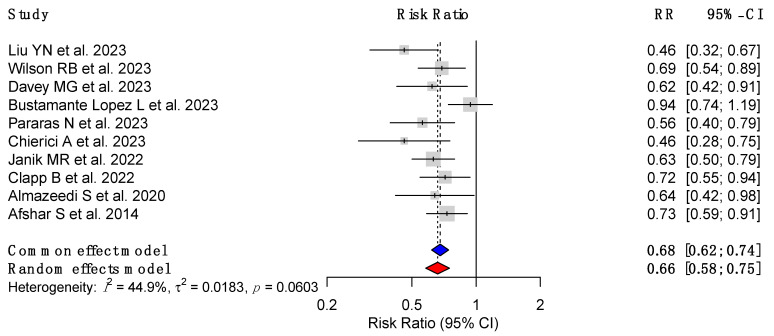
Forest plot depicting the reduction in the risk of colorectal cancer (CRC) following metabolic bariatric surgery (MBS), based on the results from included studies [7,18,19,20,21,22,23,24,25,27].

Heterogeneity (I^2^) varied across studies. High heterogeneity was noted in Liu et al. [18] (97.8%) and Davey et al. [20] (99%), likely reflecting differences in study populations, methodologies, or follow-up durations. Conversely, Almazeedi et al. (2020) [25] demonstrated minimal heterogeneity (I^2^ = 5%), indicating uniform findings in a smaller Kuwaiti cohort. Collectively, these data highlight a consistent protective association with the pooled effect size of 0.68 (95% CI: 0.62–0.74) between MBS and CRC risk reduction across diverse populations and study designs (Figure 2).

### 3.4. Sensitivity Analysis of CRC Risk in Patients with MBS

**Figure 3:**
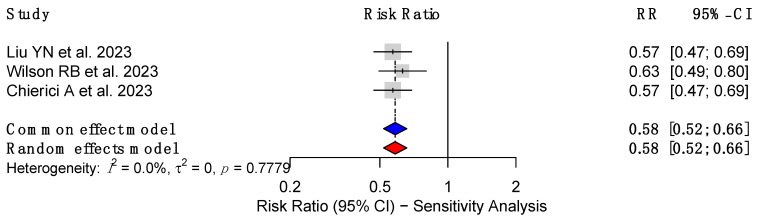
Forest plot illustrating the results of the sensitivity analysis for studies examining the risk reduction in colorectal cancer following MBS [18,19,23].

Sensitivity analyses confirmed the robustness of the findings. Liu et al. (2023) [18] and Chierici et al. (2023) [23] consistently reported protective associations with recalculated RRs of 0.57 (95% CI: 0.47–0.69, *p* = 0.0001) and moderate heterogeneity (I^2^ = 75%). Similarly, Wilson et al. (2023) [19] observed an RR of 0.63 (95% CI: 0.50–0.81, *p* = 0.0002) despite higher heterogeneity (I^2^ = 91%). Almazeedi et al. (2020) [25] confirmed the protective role of MBS with an RR of 0.53 (*p* < 0.001) in the Kuwaiti population, while Afshar et al. (2014) [27] reported an RR of 0.58 (95% CI: 0.35–0.97). Despite some heterogeneity across studies, the sensitivity analyses consistently validated the protective role of MBS in reducing CRC risk with a pooled effect size of 0.58 (95% CI: 0.52–0.66) (Figure 3).

### 3.5. Effect of MBS on Colon and Rectal Cancer Risk

#### 3.5.1. Effect on Colon Cancer Risk

**Figure 4:**
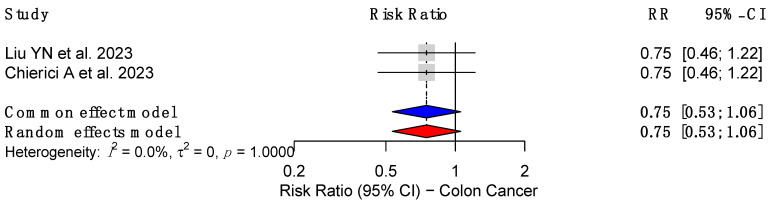
Forest plot showing the association between metabolic bariatric surgery (MBS) and the risk of colon cancer [18,23].

Across the studies analyzing colon cancer risk, a consistent association with reduced RR was observed. Liu YN et al. (2023) [18] and Chierici A et al. (2023) [23] both reported an RR of 0.75 (95% CI: 0.46–1.21, *p* = 0.2444) with a high heterogeneity of 89%. These results suggest a potential protective effect of MBS against colon cancer with a pooled effect size of 0.75 (95% CI: 0.53–1.06), albeit with considerable variability across populations (Figure 4).

#### 3.5.2. Effect of MBS on Rectal Cancer Risk

**Figure 5:**
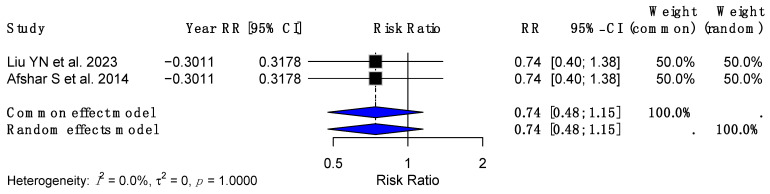
Forest plot representing the association between metabolic bariatric surgery (MBS) and the risk of rectal cancer [18,27].

For rectal cancer, a reduced RR of 0.74 (95% CI: 0.40–1.39, *p* = 0.3523) was reported, with a heterogeneity (I^2^) of 87%. This suggests a potential trend toward risk reduction; however, the wide confidence interval and lack of statistical significance highlight the uncertainty in the effect. Notably, the analysis was limited by fewer studies reporting data specific to rectal cancer, as only Chierici A et al. (2023) [23] and Liu YN et al. (2023) [18] provided relevant results. A pooled effect size of 0.74 (95% CI: 0.48–1.15) was observed (Figure 5).

### 3.6. Sex-Specific Analysis of CRC Risk Reduction Following MBS

The sex-specific analysis revealed distinct trends in the effect of MBS on CRC risk in male and female populations. For males, the pooled RR ranged from 0.65 (95% CI: 0.43–0.96, *p* = 0.03) as reported by Wilson RB et al. (2023) [19] to 0.74 (95% CI: 0.43–1.28, *p* = 0.2798) in Liu YN et al. (2023) [18]. While the overall RR indicated a protective association, the wide confidence intervals and heterogeneity (I^2^ = 96%) suggest variability in the observed effect across populations. Wilson RB et al.’s study notably demonstrated statistical significance, indicating a consistent protective effect of MBS against CRC in males.

**Figure 6:**
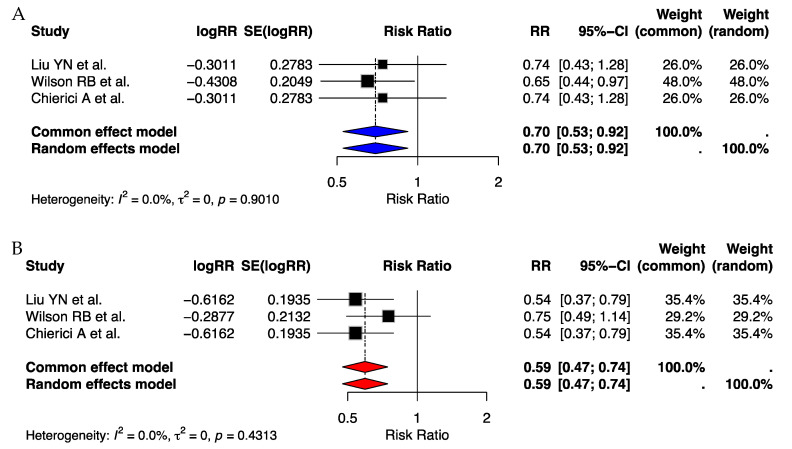
Forest plot illustrating the association between metabolic bariatric surgery (MBS) and the risk of colorectal cancer [18,19,23]. (**A**) shows the results for the male population, while (**B**) presents the findings for the female population.

In contrast, the effect of MBS on CRC risk in females demonstrated a consistent trend towards risk reduction. Liu YN et al. (2023) [18] reported an RR of 0.54 (95% CI: 0.37–0.79, *p* = 0.0014) with moderate heterogeneity (I^2^ = 90%), signifying a significant protective association. Similarly, Wilson RB et al. (2023) [19] and Chierici A et al. [23] observed an RR of 0.75 (95% CI: 0.49–1.14, *p* = 0.17), although the result was not statistically significant. The overall trend across studies suggests a possible sex-specific difference, warranting further investigations (Figure 6).

### 3.7. Procedure-Specific Analysis of CRC Risk Reduction Following MBS

The analysis of the effect of various MBS procedures on CRC risk highlighted differences in their impact. For gastric banding, the pooled RR was 0.513 (95% CI: 0.336–0.818), indicating a significant reduction in CRC risk, as reported by Davey MG et al. (2023) [20].

In contrast, sleeve gastrectomy demonstrated a pooled RR of 0.484 (95% CI: 0.307–0.763, *p* < 0.001) with considerable heterogeneity (I^2^ = 77.81%). This finding underscores a significant protective effect of sleeve gastrectomy, although the variability across studies suggests the influence of study design or population differences.

For Roux-en-Y gastric bypass (RYGB), the pooled RR was 0.64 (95% CI: 0.47–0.61, *p* = 0.05) with moderate heterogeneity (I^2^ = 64.22%). This result also highlights a protective association with CRC risk, albeit slightly less pronounced than sleeve gastrectomy.

These findings reveal procedure-specific differences in the magnitude of CRC risk reduction, with sleeve gastrectomy exhibiting the most substantial protective effect (Table 2).

### 3.8. Publication Bias

**Figure 7:**
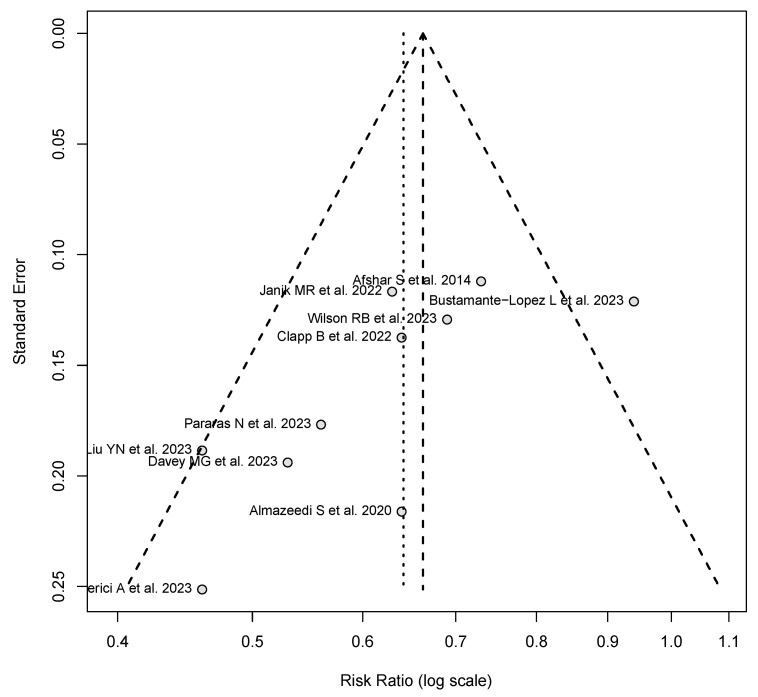
Funnel plot assessing publication bias in the meta-analysis [7,18,19,20,21,22,23,24,25,27].

The funnel plot demonstrated an asymmetric distribution of study estimates, suggesting potential publication bias. The included studies varied in standard error, with smaller studies displaying a wider dispersion around the central estimate. Egger’s test yielded a statistically significant result (*p* < 0.05), further supporting the presence of publication bias. These findings indicate that the reported effect size may be influenced by selective reporting, warranting cautious interpretation of the overall meta-analytic conclusions (Figure 7).

### 3.9. AMSTAR 2 Evaluation

The AMSTAR 2 evaluation of the included systematic reviews highlighted a wide range of methodological quality, as detailed in Table 2. Among the ten SR and MA assessed, one review was rated as “High Quality,” indicating compliance with all critical and non-critical criteria [7]. This review demonstrated rigorous adherence to AMSTAR 2 standards, including a well-defined protocol, comprehensive search strategy, transparent reporting of included and excluded studies, and appropriate synthesis methods.

One review was rated as “Moderate Quality,” with minor non-critical weaknesses identified, including partial adherence to search strategy rigor and limited justification for study exclusions [24].

Six reviews were classified as “Low Quality,” primarily due to critical flaws such as the absence of a list of excluded studies and incomplete adherence to risk-of-bias assessments [19,21,22,23,25,27]. Additionally, two reviews were categorized as “Critically Low Quality,” as they failed to meet multiple critical AMSTAR 2 criteria, such as the lack of protocol registration and inadequate reporting of heterogeneity [18,20] (Table S4).

Notably, reviews rated as “Low Quality” and “Critically Low Quality” frequently shared deficiencies in Items 4, 7, and 9, which pertain to the documentation of excluded studies, adequacy of search strategies, and methods used to assess the quality of included studies (Table 3).

These findings underscore the heterogeneity in the methodological rigor of the systematic reviews included in this umbrella review. While the high-quality review provides robust evidence, the prevalence of low- and critically low-quality reviews emphasizes the need for future research to address critical methodological shortcomings to improve the reliability of evidence synthesis in the field.

## 4. Discussion

The global burden of obesity has reached unprecedented levels, highlighting its significance as a critical public health concern. In 2022, approximately 2.5 billion adults aged 18 years and older were classified as overweight, with over 890 million individuals living with obesity, accounting for 43% of adults globally, a significant rise from 25% in 1990 [1]. Regional disparities are striking, with prevalence ranging from 31% in the WHO South-East Asia and African Regions to 67% in the Region of the Americas [28].

Metabolic and bariatric surgery has emerged as a transformative surgical intervention for the management of severe obesity and its associated comorbidities, including type 2 diabetes, hypertension, and dyslipidemia [29]. Beyond its metabolic benefits, MBS has been increasingly recognized for its potential to mitigate obesity-related cancer risks, including colorectal cancer, as seen in this umbrella review [7,18,19,20,22,23,24,25,27] (Figure 2 and Figure 3).

Our objective was to elucidate the association between patients undergoing metabolic bariatric surgery and its effect on developing colorectal cancer. This umbrella review included a total of 10 meta-analyses that ranged from the years 2020 to 2023, with a combined sample size of 53,452,658 patients. Our findings emphasize a risk reduction in colorectal cancer (pooled effect size: 0.68; 95% CI: 0.62–0.74) in those who underwent MBS, after a variable follow-up time ranging from 3 to more than 20 years (Figure 2 and Figure 3). More specifically, a greater risk reduction was seen in the female population, with a pooled effect size of 0.54 (95% CI: 0.41–0.71), in comparison to the male population (pooled effect size of 0.74, 95% CI: 0.50–1.09). Additionally, procedure specific discrepancies were noted, and a trend towards greater colorectal cancer risk reduction in those who underwent sleeve gastrectomy or RYGB, in comparison to gastric banding (Table 2).

Not surprisingly, our findings support the potential impact and benefits that MBS has been associated with [30]. An umbrella review of 204 systematic reviews and meta-analyses conducted by Kyrgiou M et al. (2017) highlighted the relationship between excess body fat and cancer risk, demonstrating a 9% increased risk (RR 1.09, 95% CI 1.06–1.13) of rectal cancer in men for every 5 kg/m^2^ rise in BMI [31]. This elevated risk extends to colorectal cancer prognosis, where obesity has been associated with more advanced disease at presentation, including stage II or III cancer and more extensive lymph node involvement (N > 3) [32]. Pre-diagnostic obesity has also been linked to worse outcomes, including a higher risk of disease-specific mortality and reduced overall survival [8,32,33]. Notably, a meta-analysis of 16 prospective cohort studies involving 58,917 patients found that individuals with pre-known obesity increased colorectal cancer-specific mortality by 22% and all-cause mortality by 25%. Post-diagnosis obesity, with a BMI of ≥35, further elevated the risk of all-cause mortality by 13% [34,35].

Regardless of conflicting findings between the latter, the well-known association between obesity and cancer supports the plausibility that MBS may not only be the most effective treatment for obesity and its metabolic components [12], but that it may confer protection when it comes to obesity-related cancers, like colorectal cancer. MBS is hypothesized to reduce the risk of cancer by multiple mechanisms. Primarily it is noted that MBS leads to greater and more sustained weight loss over time than diet, lifestyle, medications, and/or a mix of the latter [36].

The mechanisms underlying the protective effects of MBS against CRC remain multifactorial and complex. They may involve sustained weight loss, improvements in insulin sensitivity, reduction in chronic inflammation, and favorable changes in gut microbiota composition.

In contrast to our findings, a study by Tao W. et al. (2019) [37] observed an increased risk of colon cancer in the 10–14 year follow-up period post-MBS (HR: 1.55, 95% CI: 1.04–2.31) and rectal cancer in the 20+ year follow-up period post-MBS (HR: 2.11, 95% CI: 1.03–4.32). Our review found a potential protective effect of MBS against colon cancer, with a pooled RR of 0.75 (95% CI: 0.46–1.21, *p* = 0.2444) and a reduced risk for rectal cancer (RR: 0.74, 95% CI: 0.40–1.39, *p* = 0.3523) [18,23]. However, both analyses share significant heterogeneity (89% for colon cancer and 87% for rectal cancer in our review), therefore limiting its external validity. The latter discrepancies may reflect differences in follow-up durations, underlying population characteristics, type of procedure, or cancer surveillance practices. Notably, the increased risk observed by Tao W. et al. (2019) in long-term follow-up warrants further investigation, as it may suggest delayed effects of MBS on CRC risk. These findings underscore the complexity of the relationship between MBS and CRC and emphasize the need for robust, longitudinal studies to reconcile these divergent outcomes and provide greater clarity on the long-term effects of MBS.

Similarly, Bustamante-Lopez et al. [21], one of the studies included in our umbrella review, did not identify a decreased risk of early-onset CRC following MBS (RR: 0.94, 95% CI: 0.74–1.19). Their meta-analysis comprised five studies, predominantly from North America (3/5). Notably, as the primary objective of their study was to assess the impact of MBS specifically on early-onset CRC, their highly specific inclusion criteria may account for this being the only study in our review that did not demonstrate a reduced CRC risk post-MBS. It is important to highlight that their inclusion criteria were particularly stringent, focusing exclusively on individuals under 50 years of age with a BMI > 35 kg/m^2^ who underwent colonoscopy with a strict follow-up interval (<5 years). Furthermore, the study assessed only the overall risk of CRC without differentiating between colonic and rectal cancers. Given its distinct objective, highly selective population, and methodological approach, this study presents unique findings within the broader body of evidence.

Furthermore, when comparing the effects of MBS in individuals with obesity patients with the risk of developing CRC, it is difficult to ignore how sex may affect this risk. For instance, Hussan H. et al. (2022) [38] investigated sex-specific correlations between obesity and CRC, noting a more pronounced association in males, concluding that males had a higher risk of CRC, particularly rectosigmoid cancer, than females after bariatric surgery (HR = 2.69, 95% CI: 1.35–5.38, *p* < 0.001), and that there was a decrease in CRC risk in females’ post-RYGB (HR = 0.40, 95%CI: 0.18–0.87, *p* = 0.02). Liu YN. et al. (2023) [18] published a significant reduction in CRC risk among individuals with obesity patients who underwent bariatric surgery, but particularly a significant 46% reduction in colorectal cancer risk observed among female patients (RR: 0.54, 95%CI: 0.37–0.79, *p* = 0.0014, I^2^ = 90%).

Notably, our findings suggest that sleeve gastrectomy (SG) demonstrates the most substantial and statistically significant protective effect, with a pooled RR of 0.484 (95% CI: 0.307–0.763), followed by Roux-en-Y gastric bypass (RYGB), with a pooled RR of 0.64 (95% CI: 0.47–0.61). In contrast, gastric banding, though associated with a trend towards a reduction in CRC risk (RR: 0.513, 95% CI: 0.336–0.818), appears less protective than SG. Importantly, these findings highlight significant heterogeneity, particularly for SG (I^2^ = 77.81%), thereby limiting its interpretation and applicability. It is important to note that gastric banding was previously amongst the most common MBS procedures and that currently RYGB and SG have replaced the latter. Therefore, an updated comparison between the most common MBS procedures not limited to SG and RYGB should be taken into consideration. When it comes to procedure-specific analysis, it is unclear whether it is the procedure’s capacity to decrease the most amount of weight, sustainable and long-term weight loss, or whether it is the restrictive or malabsorptive nature of certain procedures that most contributes to this phenomenon [36]. Unlike our findings, RYGB has been previously associated with an increase in colorectal cancer, and this has been attributed to an increased exposure to bile acids from the colon and increased colon proliferation mediated by COX-2, which are secondary to anatomical variants secondary to the surgical interventions of MBS [36].

Comparatively, our conclusions diverge from those of Chierici A. et al. [23], who reported no significant difference in CRC risk reduction between SG and RYGB (RR: 1.03, 95% CI: 0.72–1.47, *p* = 0.8713, I^2^ = 45%). The discrepancies between these findings may be attributed to differences in the selection of studies included in the meta-analyses, statistical methods, or population-specific factors. Chierici A. et al. emphasize a more uniform protective effect across procedures, contrasting with our observation of SG’s superior risk reduction. This divergence underscores the importance of analyzing procedural outcomes within the context of evolving clinical practices and patient-specific factors such as BMI, comorbidities, and postoperative follow-up protocols.

Several cohort studies have reported increased incidences of taxanes and platinum-induced neuropathy in patients with obesity [39,40]. Additionally, recent findings suggest that excess body fat is linked to a heightened risk of treatment-related cardiotoxicity [41,42].

Further studies that account for these variables are essential to reconcile these differences and establish a consensus. While our findings highlight the heterogeneity inherent in SG studies, they also suggest procedure-specific potential advantages in CRC risk mitigation, warranting further exploration. It is imperative that future studies focus on providing detailed and homogeneous definitions of their criteria for MBS, patient population demographics, pre- and postoperative BMI, procedure type, follow-up times, and screening policies with the objective of establishing more homogeneity amongst distinct studies.

Our AMSTAR 2 findings underscore the heterogeneity in the methodological rigor of the systematic reviews included in this umbrella review. While the high-quality review provides robust evidence, the prevalence of low- and critically low-quality reviews emphasizes the need for future research to address critical methodological shortcomings to improve the reliability of evidence synthesis in the field. Knowledge of the AMSTAR2 grading score and PRISMA tool can help authors mitigate low- and critically low-quality reviews and therefore leverage the clinical impact of these findings. Moreover, the implementation of this tool as part of the inclusion criteria may strengthen the associations and findings and ultimately build towards greater clarity and clinical decisions.

There are several inherent limitations to an umbrella review. Significant heterogeneity in the study designs, methods and reporting systems, follow-up durations, and outcome reporting present challenges in drawing uniform and statistically and clinically significant conclusions. Furthermore, as a “review of reviews”, this type of study is limited by the quality and divergence across distinct literature and populations. The underrepresentation of certain geographic regions and populations further restricts the generalizability of the findings, underscoring the need for broader, more inclusive research. Despite these limitations, the use of robust sensitivity analyses and the consistency of observed trends enhance the reliability and relevance of the results.

Our findings underscore the importance of addressing obesity to mitigate the burden of obesity-related cancers, particularly CRC, which is increasingly prevalent in younger populations. Minimally invasive bariatric surgery has shown promise in mitigating obesity and associated cancer risks. Further investigation is needed to better understand the mechanisms driving these associations. Investigating hormonal, metabolic, and gut microbiome alterations post-surgery is essential to understanding the pathways linking MBS to reduced cancer risk. Additionally, prospective longitudinal studies with standardized methodologies and extended follow-up durations are critical for validating and refining these findings across diverse populations. Integrating genomic analyses and biomarker research into future studies may also identify high-risk subgroups who could benefit most from MBS interventions.

Expanding the scope of studies to include broader and more inclusive cohorts will enhance the generalizability of the findings. These future directions hold the potential to improve patient outcomes, refine preventative strategies, and drive innovations in managing obesity-associated malignancies, particularly CRC. By addressing these gaps, future research can provide a more comprehensive understanding of MBS’s role in reducing cancer burden and improving global health outcomes.

## 5. Conclusions

This umbrella review consolidates current evidence on the association between MBS and CRC risk, demonstrating a consistent protective effect. Despite some degree of heterogeneity among studies, sensitivity analyses reinforce the robustness of this protective association. The findings suggest a potential risk reduction for both colon and rectal cancer, with a more pronounced effect observed in females compared to males. While certain studies indicate a lower relative risk with SG, the evidence remains inconclusive due to the limited number of direct comparative analyses between surgical procedures. Given the substantial implications of MBS on cancer incidence, morbidity, and mortality, further high-quality, long-term studies with direct procedural comparisons are imperative. These studies should aim to elucidate the mechanistic pathways underlying MBS’s protective effect, refine patient selection criteria, and optimize surgical strategies to enhance oncological outcomes and guide evidence-based clinical decision-making.

## Figures and Tables

**Table 1:** Summary of study characteristics, including authors, publication year, country of origin, number of studies included in each systematic review, and the total patient population.

Authors	Journal	Year of Publication	Country of Publication	Total Number of Studies Included	Total Number of Patients
Liu YN et al. [18]	*World Journal of Gastrointestinal Surgery*	2023	China	17	12,497,322
Wilson RB et al. [19]	*International Journal of Molecular Science*	2023	Australia	32	3,526,338
Davey MG et al. [20]	*Obesity Surgery* *The Journal of Metabolic Surgery and Allied Care*	2023	Ireland	11	6,214,682
Bustamante-Lopez L et al. [21]	*Updates in Surgery*	2023	USA	5	48,916
Pararas N et al. [22]	*International Journal of Environmental Research and Public Health*	2023	Greece	13	6,279,722
Chierici A et al. [23]	*Nutrients*	2023	France	18	12,517,893
Janik MR et al. [24]	*Surgery for Obesity and Related Diseases*	2022	Poland	13	3,233,044
Clapp B et al. [7]	*British Journal of Surgery*	2022	USA	15	947,787
Almazeedi S et al. [25]	*British Journal of Surgery*	2020	Kuwait	7	1,213,727
Afshar S et al. [27]	*Obesity Surgery* *The Journal of Metabolic Surgery and Allied Care*	2014	UK	4	105,187

**Table 2:** Pooled relative risk (RR) estimates and confidence intervals (CI) for gastric banding, sleeve gastrectomy, and Roux-en-Y gastric bypass (RYGB) in reducing colorectal cancer (CRC) risk following metabolic and bariatric surgery (MBS).

Authors	Effect Size of Gastric Band					Effect Size of Sleeve Gastrectomy					Effect Size of RYGB				
	RR	95% CI Lower	95% CI Upper	*p*-value	I^2^ (%)	RR	95% CI Lower	95% CI Upper	*p*-value	I^2^ (%)	RR	95% CI Lower	95% CI Upper	*p*-value	I^2^ (%)
Davey MG et al. [20]	0.513	0.336	0.818	-	-	0.484	0.307	0.763	-	-	-	-	-	-	-
Pararas N et al. [22]	0.77	0.48	1.22	0.27	-	0.55	0.36	0.83	<0.001	-	0.64	0.41	1.00	0.05	-
Clapp B et al. [7]	1.34	0.28	7.12	-	99.08	0.55	0.36	0.83	-	77.81	0.47	0.36	0.61	-	64.22

RR: relative risk; CI: confidence interval; RYGB: Roux-en-Y gastric bypass.

**Table 3:** AMSTAR 2 quality ratings for included systematic reviews.

AMSTAR 2 Item	Liu YN et al. [18]	Wilson RB et al. [19]	Davey MG et al. [20]	Bustamante-Lopez et al. [21]	Pararas N et al. [22]	Chierici A et al. [23]	Janik MR et al. [24]	Clapp B et al. [7]	Almazeedi S et al. [25]	Afshar S et al. [27]
1. Did the research questions and inclusion criteria for the review include the components of PICO?	YES	YES	YES	YES	YES	YES	YES	YES	YES	YES
2. Did the report of the review contain an explicit statement that the review methods were established prior to the conduct of the review and did the report justify any significant deviations from the protocol?	YES	YES	YES	YES	YES	YES	PARTIAL YES	YES	YES	YES
3. Did the review authors explain their selection of the study designs for inclusion in the review?	YES	YES	YES	YES	YES	YES	YES	YES	YES	YES
4. Did the review authors use a comprehensive literature search strategy?	PARTIAL YES	YES	PARTIAL YES	YES	YES	YES	PARTIAL YES	YES	PARTIAL YES	YES
5. Did the review authors perform study selection in duplicate?	YES	YES	NO	YES	YES	NO	YES	YES	YES	YES
6. Did the review authors perform data extraction in duplicate?	NO	YES	YES	YES	YES	NO	YES	YES	YES	YES
7. Did the review authors provide a list of excluded studies and justify the exclusions?	NO	NO	NO	NO	NO	NO	PARTIAL YES	PARTIAL YES	NO	NO
8. Did the review authors describe the included studies in adequate detail?	YES	YES	PARTIAL YES	YES	YES	YES	YES	YES	YES	PARTIAL YES
9. Did the review authors use a satisfactory technique for assessing the risk of bias (RoB) in individual studies that were included in the review?	YES	YES	PARTIAL YES	PARTIAL YES	YES	YES	PARTIAL YES	YES	PARTIAL YES	YES
10. Did the review authors report on the sources of funding for the studies included in the review?	NO	NO	NO	NO	NO	NO	NO	NO	NO	NO
11. If meta-analysis was performed, did the review authors use appropriate methods for statistical combination of results?	YES	YES	YES	YES	YES	YES	YES	YES	YES	YES
12. If meta-analysis was performed, did the review authors assess the potential impact of RoB in individual studies on the results of the meta-analysis or other evidence synthesis?	YES	YES	YES	YES	YES	YES	YES	YES	YES	YES
13. Did the review authors account for RoB in individual studies when interpreting/discussing the results of the review?	YES	YES	YES	YES	YES	YES	YES	YES	YES	YES
14. Did the review authors provide a satisfactory explanation for, and discussion of, any heterogeneity observed in the results of the review?	YES	YES	YES	YES	YES	YES	YES	YES	YES	YES
15. If they performed quantitative synthesis, did the review authors carry out an adequate investigation of publication bias (small study bias) and discuss its likely impact on the results of the review?	YES	YES	YES	YES	YES	YES	YES	YES	YES	YES
16. Did the review authors report any potential sources of conflict of interest, including any funding they received for conducting the review?	YES	YES	YES	YES	YES	YES	YES	YES	YES	YES
Final Rating	CRITICAL LOW	LOW QUALITY	CRITICAL LOW	LOW QUALITY	LOW QUALITY	LOW QUALITY	MODERATE	HIGH	LOW QUALITY	LOW QUALITY

(HIGH: systematic reviews with zero or one non-critical weakness, providing an accurate and comprehensive summary of available evidence. MODERATE: reviews with more than one non-critical weakness but no critical flaws. These reviews are generally reliable but may have limitations in comprehensiveness. LOW: reviews with at least one critical flaw, reducing confidence in their findings. CRITICALLY LOW: reviews with more than one critical flaw, rendering them unreliable for evidence synthesis).

## Data Availability

The datasets generated and/or analyzed during this study are not publicly available but may be obtained from the corresponding author upon reasonable request.

## Supplementary Materials

The following supporting information can be downloaded at: https://www.mdpi.com/article/10.3390/cancers17040670/s1, Table S1: Search terms for the meta-analysis in PubMed, Scopus, Web of Science, and Science Direct databases. Table S2: Articles that were fully read and excluded. Table S3: Overlapping articles with the included studies. Table S4: AMSTAR 2 quality ratings for included systematic reviews.
