# Supplementary material for: Outcomes of Metabolic and Bariatric Surgery in Populations with Obesity and Their Risk of Developing Colorectal Cancer: Where Do We Stand? An Umbrella Review on Behalf of TROGSS—The Robotic Global Surgical Society
Source: Cancers (Basel). 2025 Feb 17;17(4):670. doi: 10.3390/cancers17040670 (PMC11853171; doi:10.3390/cancers17040670)
Supplement: Supplementary file 1 [file cancers-17-00670-s001.zip › cancers-3382142-supplementary.pdf]

Table S1. Search terms for the meta-analysis in PubMed, Scopus, Web of Science, and Science Direct databases.

Database	Search terms
PubMed (n=232) SR = 46	("metabolic bariatric surgery" OR "bariatric surgery" OR "weight loss surgery" OR "obesity surgery" OR "gastric bypass" OR "sleeve gastrectomy" OR "roux-en-y gastric bypass") AND ("colorectal cancer" OR "colon cancer" OR "rectal cancer" OR "colorectal neoplasm" OR "colorectal carcinoma" OR "CRC")
Web of Science (n=306) Review Articles = 93	TS= ("metabolic bariatric surgery" OR "bariatric surgery" OR "weight loss surgery" OR "obesity surgery" OR "gastric bypass" OR "sleeve gastrectomy" OR "roux-en-y gastric bypass") AND TS= ("colorectal cancer" OR "colon cancer" OR "rectal cancer" OR "colorectal neoplasm" OR "colorectal carcinoma" OR "CRC")
Scopus (n=471) Review Articles =127	TITLE-ABS-KEY ("metabolic bariatric surgery" OR "bariatric surgery" OR "weight loss surgery" OR "obesity surgery" OR "gastric bypass" OR "sleeve gastrectomy" OR "roux-en-y gastric bypass") AND TITLE-ABS-KEY ("colorectal cancer" OR "colon cancer" OR "rectal cancer" OR "colorectal neoplasm" OR "colorectal carcinoma" OR "CRC")
Science Direct (n=1000)	Title, abstract, keywords: (cancer OR Neoplasm OR tumor) AND (colorectal OR rectal OR colon)
Embase (n=839) Review Articles = 70	('metabolic bariatric surgery' OR 'bariatric surgery'/exp OR 'bariatric surgery' OR 'weight loss surgery'/exp OR 'weight loss surgery' OR 'obesity surgery'/exp OR 'obesity surgery' OR 'gastric bypass'/exp OR 'gastric bypass' OR 'sleeve gastrectomy'/exp OR 'sleeve gastrectomy' OR 'roux-en-y gastric bypass'/exp OR 'roux-en-y gastric bypass') AND ('colorectal cancer'/exp OR 'colorectal cancer' OR 'colon cancer'/exp OR 'colon cancer' OR 'rectal cancer'/exp OR 'rectal cancer' OR 'colorectal neoplasm'/exp OR 'colorectal neoplasm' OR 'colorectal carcinoma'/exp OR 'colorectal carcinoma' OR 'crc')

Table S2. Articles that were fully read and excluded.

Article Name	Reason for exclusion
Michal Janik Warsaw, Przemyslaw Sroczynski Warsaw, Benjamin Clapp, Omar Ghanem Rochester. The effect of bariatric surgery on reducing the risk of colorectal cancer: a meta-analysis, Surgery for Obesity and Related Diseases,Volume 18, Issue 8, Supplement, 2022,Page S4,ISSN 1550-7289, https://doi.org/10.1016/j.soard.2022.06.029	**REPEATED**
Elabd, R., Almazeedi, S., Al-Sabah, S. 24th IFSO World Congress. *OBES SURG* 29 (Suppl 5), 347–1720 (2019). https://doi.org/10.1007/s11695-019-04101-1	**ABSTRACT ONLY**
Chang S.H., Jang J., Popov V.Bariatric Surgery Is Associated with Increased Risk Of Colorectal Cancer: A Systematic Meta-Analysis. Gastroenterology 2019 156:6 (S-1167) S1. 10.1016/S0016-5085(19)39887-7	**ABSTRACT ONLY**
Lee S., Chirumamilla S.,Okolocha C., Sanni A.Scientific Session of the 16th World Congress of Endoscopic Surgery, Jointly Hosted by Society of American Gastrointestinal and Endoscopic Surgeons (SAGES) & Canadian Association of General Surgeons (CAGS), Seattle, Washington, USA, 11–14 April 2018: Poster Abstracts. *Surg Endosc* 32 (Suppl 1), 130–359 (2018). https://doi.org/10.1007/s00464-018-6121-4	**ABSTRACT ONLY**
Yang XW, Li PZ, Zhu LY, Zhu S. Retraction Note: Effects of Bariatric Surgery on Incidence of Obesity-Related Cancers: A Meta-Analysis. Med Sci Monit. 2016 May 24;22:1751. doi: 10.12659/msm.899711. PMID: 27215479; PMCID: PMC4917316	**RETRACTED**

Table S3. Overlapping Articles with the included studies.

Article names	Follow up (yrs)	Liu YN et al. [18]	Wilson RB et al. [19]	Davey MG et al. [20]	Bustamante-Lopez L et al. [21]	Pararas N et al. [22]	Chierici A et al. [23]	Janik MR et al. [24]	Clapp B et al. [7]	Almazeedi S et al. [25]	Afshar S et al. [27]
Adams et al,2009	12.5										
Christou et al, 2008	5										
McCawley et al, 2009	-										
Derogar et al, 2013	10										
Aravani et al, 2018	3										
Mackenzie et al, 2018	4.6										
Khalid et al, 2022	5										
Kwak et al, 2019	7.8										
Schauer et al, 2019	3.5										
Tao et al, 2020	3.1										
Bailly et al, 2020	5.7										
Tsui et al, 2020	-										
Taube et al, 2021	22.2										
Aminian et al, 2022	6.1										
Desai et al, 2022	-										
Hussan et al, 2022	3										
Lazzati et al, 2022	5.7										
Cicciorriccio et al, 2021	4.3										
Gomez et al, 2020	3.5										

Rustgi et al, 2021	-										
Yang et al, 2016	-										
Martet-Ouda et al,2017	3.54										
Njei et al , 2018	5.33										
Kwak et al, 2020	7.1										
Botero-Fonnegra et al, 2022	-										
Wei et al, 2021	3.08										
Andalib et al , 2021	7.6										
Droney et al, 2021	-										
Hussan et al, 2020	60 mo										

Table S4. AMSTAR 2 quality ratings for included systematic reviews.

Study	AMSTAR 2 Quality Rating	NOTES
**Ying-Ning Liu [18]**	CRITICAL LOW QUALITY	Item 4 "Partial YES" answer is still considered a critical flaw. It does not provided a list of all potentially relevant studies that were read in full text form but excluded from the review
**S. Almazeedi [25]**	LOW QUALITY	Item 4 "Partial YES" answer is still considered a critical flaw
**Robert B. Wilson [19]**	LOW QUALITY	It does not provided a list of all potentially relevant studies that were read in full text form but excluded from the review
**Matthew G. Davey [20]**	CRITICAL LOW QUALITY	Item 4 "Partial YES" answer is still considered a critical flaw. It does not provided a list of all potentially relevant studies that were read in full text form but excluded from the review
**Michał R. Janik [24]**	MODERATE QUALITY	While Items 9 and 14 are clearly designated as non-critical weaknesses, Item 7 with a Partial Yes is usually treated as a non-critical weakness as well, depending on the severity of the shortcoming in the search strategy.
**Benjamin Clapp [7]**	HIGH QUALITY	While Items 9 and 14 are clearly designated as non-critical weaknesses, Item 7 with a Partial Yes is usually treated as a non-critical weakness as well, depending on the severity of the shortcoming in the search strategy.
**L. Bustamante-Lopez [21]**	LOW QUALITY	Regarles one non-critical weakness, it has one critical flaw related to not providing a list of all potentially relevant studies that were read in full text form but excluded from the review
**Sorena Afshar [27]**	LOW QUALITY	It does not provided a list of all potentially relevant studies that were read in full text form but excluded from the review
**Nikolaos Pararas[22]**	LOW QUALITY	It does not provided a list of all potentially relevant studies that were read in full text form but excluded from the review
**Andrea Chierici [23]**	LOW QUALITY	It does not provided a list of all potentially relevant studies that were read in full text form but excluded from the review
